# Clinical presentation of air leak in an infant with undiagnosed cystic fibrosis: a case report

**DOI:** 10.1186/s13256-015-0620-x

**Published:** 2015-07-11

**Authors:** Mohammed M. Al-Balawi, Khalid Al-Mobaireek, Wadha Alotaibi, Abdullah Al-Shamrani, Khalid S. Ahmad, Suhail Al-Saleh

**Affiliations:** 1 King Fahad Medical City, P.O. Box 59046, Riyadh, 11525 Kingdom of Saudi Arabia; 2 The Hospital for Sick Children, 555 University Avenue, Toronto, M5G 1X8, ON Canada

**Keywords:** Cystic fibrosis, Infants, Pediatrics, Pneumomediastinum, Pseudo-Bartter’s, Tracheal tear

## Abstract

**Introduction:**

Air leak is a well-recognized complication of advanced cystic fibrosis in older children and adults but is extremely rare in infants. To the best of our knowledge, this is the youngest reported pediatric case of an air leak from a major airway.

**Case presentation:**

A 4-month-old Yamani baby girl with a family history of cystic fibrosis initially presented with a history of a persistent paroxysmal cough for 3 weeks and vomiting for 1 week. Laboratory evaluation indicated pseudo-Bartter’s syndrome. Imaging showed a tracheal tear with pneumomediastinum and subcutaneous emphysema that was treated conservatively.

**Conclusions:**

This case highlights the possibility of air leak in the population of young patients with cystic fibrosis and it shows a successful conservative management of tracheal tear. Physicians should consider cystic fibrosis in infants presenting with air leak.

## Introduction

Pulmonary air leak is defined as an escape of air from normal air spaces. Air leak is a well-recognized complication of advanced cystic fibrosis (CF) in older children and adults [1], with a reported incidence of 4.6 to 6/1000/year [2, 3]. We describe a 4-month-old baby girl with both an air leak from a major airway and pseudo-Bartter’s syndrome as an initial presentation of CF that was treated conservatively. Our aim in this report is to highlight the possibility of air leak in the population of young patients with CF and to report a successful conservative management of tracheal injury in the pediatric population.

## Case presentation

A 4-month-old baby girl was admitted to our pediatric intensive care unit with acute renal failure and respiratory distress.

She was a fifth-born to consanguineous parents, born full term after an unremarkable pregnancy. Her birth weight was 4kg, and meconium was passed on the first day of life. She had a 14-year-old sister diagnosed with CF at 1 year of age. The parents were concerned regarding the salty taste of their daughter’s skin and an offensive stool odor since birth. They also noticed her stools becoming oily and bulky by 3 months of age, but she was not investigated for CF.

The patient had a persistent paroxysmal cough for 3 weeks that progressed as she developed shortness of breath and tachypnea. She also had a 1 week history of vomiting, lethargy and decreased oral intake. Her parents sought medical advice at private clinics, and bronchodilators and antibiotics were prescribed, without improvement. Three days prior to admission, the patient’s sister fell on the patient’s neck and chest; the next day, swelling was noticed on the patient’s neck.

During the initial examination, the patient was found to be irritable in severe distress, tachypneic, tachycardic with normal blood pressure, and severely dehydrated. She had palpable subcutaneous crepitation over her neck, extending to the anterior chest wall. There was intercostal and subcostal retraction; on auscultation, air entry was decreased with the bilateral presence of crepitation.

**Fig. 1:**
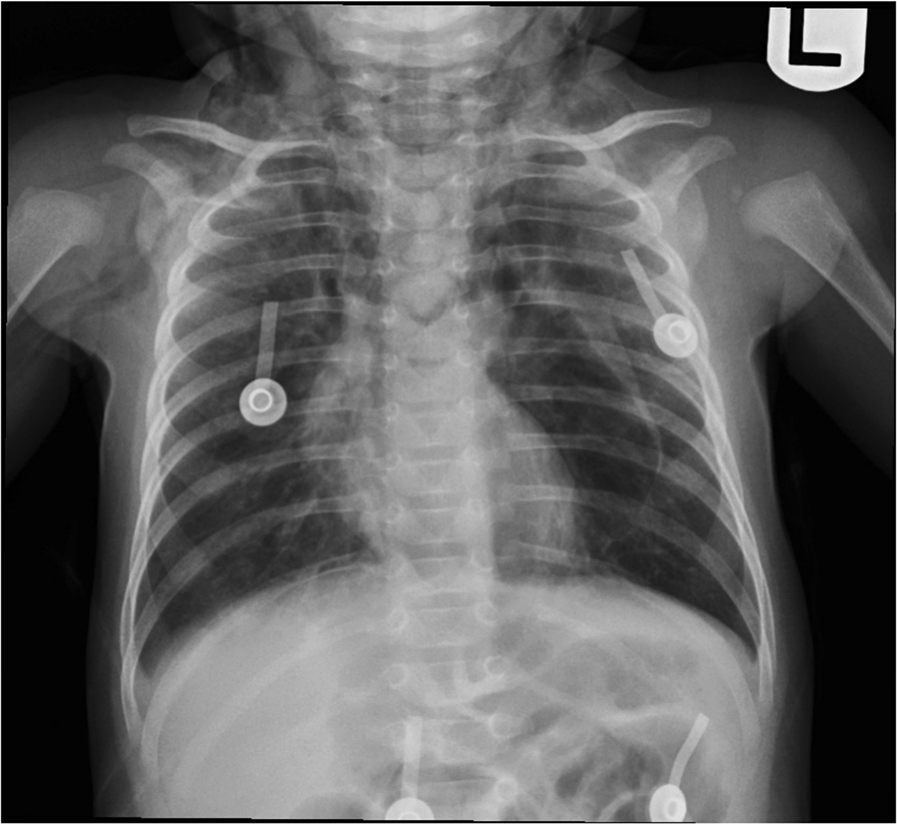
Chest X-ray. Evidence of the pneumomediastinum and soft tissue emphysema extends to the root of the neck. The lungs demonstrate bilateral heterogeneous opacities

**Fig. 2:**
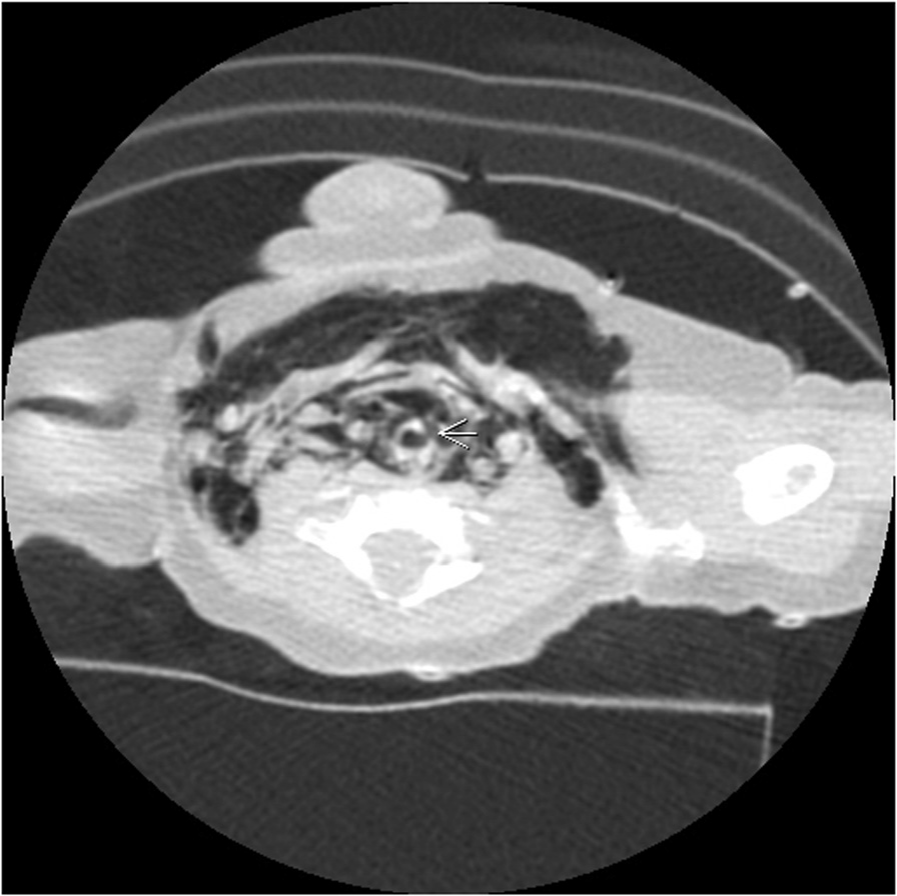
Computed tomography of the neck. Arrow pointing to a defect involving the left lateral wall of upper trachea with associated extensive pneumomediastinum and subcutaneous emphysema

A basic workup revealed metabolic alkalosis in capillary blood gas (pH 7.48; partial pressure of oxygen in arterial blood, PaO_2_, 88mmol/L; partial pressure of carbon dioxide, PCO_2_, 48mmol/L; bicarbonate, HCO_3_, 35mmol/L), hypokalemia (potassium 3mmol/L), hyponatremia (sodium 125mmol/L), hypochloremia (chloride 61mmol/L) and renal impairment with high urea (15.9mmol/L) and creatinine (155mmol/L) and low urine chloride (<20mmol/L). In addition, a normal calcium/creatinine ratio was detected in her urine (0.107mol/1mol), and a septic workup was negative. A chest X-ray (Fig. 1) showed pneumomediastinum and subcutaneous emphysema. In addition to oxygen provided by a face mask, she was given two intravenous boluses of 0.9% saline (20ml/kg each), followed by maintenance intravenous infusion of 0.45% saline with potassium chloride supplementation. Ceftriaxone and clindamycin were started intravenously. A computed tomography (CT) scan of her neck (Fig. 2) showed a tracheal tear with pneumomediastinum.

During admission, she significantly improved regarding hydration, activity, and oral intake. Electrolyte abnormalities were corrected. Her tracheal tear was managed conservatively. The neck swelling disappeared, and a subsequent X-ray and CT scan 1 week later showed complete resolution of the air leak. A sweat chloride test had high results (95mmol/L) (conductance method), and a *cystic fibrosis transmembrane conductance regulator* (*CFTR*) gene mutation test was performed. She was started on pancreatic enzymes, a vitamin supplement, a salt supplement, and regular chest physiotherapy. After completing a course of antibiotics, she was discharged in stable condition. The *CF* gene was later reported to contain a homozygous mutation (c.2988 + 1G > A; IVS18 + 1G > A).

## Discussion

Our case is the first case of CF presenting with a major airway leak in a very young patient. This indicates the possibility of air leak presentation in very young patients with CF. It is also the second case of CF to initially present with an airway leak in addition to pseudo-Bartter’s syndrome [4].

Pulmonary air leak is defined as an escape of air from normal air spaces. Depending on the location to which the air escapes, pneumothorax, pneumomediastinum, pneumopericardium, pulmonary interstitial emphysema, and subcutaneous emphysema can occur. Air leak is a well-recognized complication of CF that usually occurs in older children and adults with advanced disease, but this complication is extremely rare as an initial presentation of CF and in the very young [1–3]. Although there have been two reported cases of CF presented at 1 and 4 months of age with pneumothorax, none was related to a leak from a major airway such as the trachea in our case [5, 6].

Tracheal tear is known to follow neck trauma, including penetrating and blunt injury, or to be an iatrogenic complication of intubation [7–9]. Spontaneous air leak due to tracheal tear is extremely uncommon but has been reported in pediatric patients after excessive coughing or vomiting [10, 11]. Our case was likely spontaneous, related to a history of severe cough and vomiting. However, due to an additional history of possible injury, traumatic air leak cannot be excluded.

The association between pseudo-Bartter’s syndrome and CF is well known and has been reported to be a presenting feature of CF [12–14]. The pathophysiology involves a loss of salt in sweat. In our case, this syndrome was attributed to multiple factors, including the hot climate and the patient’s poor nutrition, current illness and fever, and limited sodium intake, as she was exclusively breast fed. A promising result has been reported on conservative management of pediatric tracheal injury [15]. In this case conservative management was successful.

## Conclusions

CF can present with air leak from a major airway in infants as a result of a persistent cough, vomiting or trauma. Physicians should keep CF in their differential diagnosis of infants presenting with air leak. Air leak also should be considered in young children who are known to have CF and who present with a new onset of respiratory distress. A conservative management approach to tracheal injury can be considered.

## Consent

Written informed consent was obtained from the patient’s parent for publication of this case report and the accompanying images. A copy of the written consent is available for review by the Editor-in-Chief of the journal.

## Abbreviations

CF: Cystic fibrosis
CT: Computed tomography
